# *My Migraine Voice* survey: disease impact on healthcare resource utilization, personal and working life in Finland

**DOI:** 10.1186/s10194-020-01185-4

**Published:** 2020-09-29

**Authors:** Marja-Liisa Sumelahti, Markku Sumanen, Merika S. Sumanen, Samuli Tuominen, Johanna Vikkula, Sanna M. Honkala, Stina Rosqvist, Minna A. Korolainen

**Affiliations:** 1 Faculty of Medicine and Health Technology, Tampere University, Tampere, Finland; 2 Medaffcon Oy, Espoo, Finland; 3 Novartis Finland Oy, Espoo, Finland; 4 Present address: Orion Pharma, Orion Corporation, Espoo, Finland

**Keywords:** Migraine, Burden, Finland, Work productivity, Quality of life, Healthcare resource utilization

## Abstract

**Background:**

A global *My Migraine Voice* survey was conducted in 31 countries among 11,266 adults who suffered from ≥4 monthly migraine days (MMD). The aim of this retrospective observational survey-based study was to analyse the country specific results in Finland in order to understand the impact of migraine based on disease severity.

**Methods:**

The included participants (3%, *n* = 338/11,266) were stratified by mean MMDs into 4 ≤ MMD < 8 (*n* = 133), 8 ≤ MMD < 15 (*n* = 139) and MMD ≥ 15 (*n* = 66) subgroups. Comorbidities, migraine-related emotional burden and impact on daily living and work productivity and activity impairment (WPAI) were assessed. Subgroup analysis on healthcare resource utilization (HCRU) due to migraine was assessed by visits to healthcare practitioners (HCPs) during the past 6 months and by hospitalizations and emergency room (ER) visits during the past 12 months. The group difference was tested using the one-way ANOVA and for categorical variables using the Chi-squared test. The association between HCRU and MMD and number of comorbidities was assessed using negative binomial regression analysis.

**Results:**

Mean age was 44 years, 93% were women and 67% (*n* = 227) were employed. Chronic migraine (CM, MMD ≥ 15) was reported in 19.5% of the respondents. The negative impact on daily functioning and emotional burden increased significantly by migraine frequency. Mean number of comorbidities was 2.4, and mean number of HCP visits during the previous 6 months was 5.9. Increase in migraine frequency and comorbidities was associated with higher HCRU. Eighty-eight percent of the respondents reported negative impact on working life and 52% experienced overall work productivity impairment. Over previous month, the mean number of missed working days for all respondents was 2.8 days of which 54% were paid sick leave days, and in CM up to 6.0 days and 30%, respectively. Both absenteeism and presenteeism were higher in the CM group.

**Conclusions:**

The emotional and functional burden was high, and the societal burden increased by frequency and severity of migraine, as shown by higher HCRU and reduced work productivity. There is a need to improve quality of care in migraine and improve migraine management related issues in both healthcare and society in Finland.

## Background

The estimated global prevalence of migraine is 10–15% and prevalence of chronic migraine is 1.4–2.2% [1, 2]. Migraine is a disabling neurological disorder that places a significant burden on the individuals but also on their family members and the whole society [3]. The impact of migraine extends beyond the physical pain of a migraine attack and it can substantially affect multiple aspects of life including day-to-day functioning, overall quality of life (QoL), emotional and social aspects such as family, work and social relationships [3, 4].

Migraine can be broadly classified by attack frequency into episodic migraine (EM) and chronic migraine (CM) according to the International Classification of Headache Disorders, 3rd edition (ICHD-3) [5]. EM is defined as fewer than 15 monthly migraine days (MMD), and CM as 15 or more days with headache, of which ≥8 are considered migraine days. The classification has been challenged by a recent study arguing that patients suffering from high frequency migraine (MMD ≥8) should be considered to have CM [6]. Regardless of EM or CM, the disability and burden of migraine increases along with increasing headache frequency [7, 8], and increasing number of migraine days equally enhances the risk for chronification of migraine [9].

Surveys across European and other countries have shown the impact of migraine on work, healthcare resource utilization (HCRU) and QoL, among other domains [10–15]. So far, *My Migraine Voice* survey conducted in 2017–2018 is the only survey revealing the global impact of migraine, including Finland [16]. There has been a paucity of data for the spectrum of migraine impact in the Nordic countries. Earlier studies from Finland have shown an almost 2-fold increase in healthcare visits and sick leave days when compared to age-matched counterparts [17–19]. In Sweden, a recent national patient organization survey showed a correlation between the loss in yearly quality adjusted life-years (QALYs) and increasing number of migraine days indicating that productivity loss represented a significant part of costs in migraine [20].

The aim of the present study was to focus on domains assessing the burden of migraine among Finnish *My Migraine Voice* survey respondents. In order to further understand the burden of migraine in a Finnish sub cohort, disease severity was assessed as MMD frequency and impact of reported comorbidities.

## Methods

*My Migraine Voice* was a cross-sectional multi-country online survey conducted in 31 countries from September 2017 to February 2018 [16]. The Finnish participants were recruited via the Finnish Migraine Association (Patient Advocacy Group, PAG) and existing online panels from GfK Health (Growth from Knowledge, https://www.gfk.com/). Informed consent was obtained prior to the survey. Survey data were handled confidentially, and anonymity of the participants was maintained throughout the study. Ethics committee review was not required due to the research format of the study.

The Finnish cohort consisted of 345 respondents who were determined as eligible based on their responses to the screening questions. Inclusion criteria were self-reported physician diagnosed migraine based on the ICHD-3 criteria, frequency of ≥4 monthly migraine days over the previous 3 months and age > 18 years. Respondents without migraine diagnosis (*n* = 7) were excluded from the current data-analysis.

### Study design

This was a global, retrospective observational survey-based study, which consisted of closed questions. The qualitative survey was performed using online bulletin boards (OBBs) to identify the key issues relating to the professional as well as daily life experienced by individuals living with migraine. The details of the OBB survey methods has been previously published [21, 22]. The final survey comprised of 87 questions which included five country-specific questions. The questionnaire was developed by a multi-professional steering committee, consisting of migraine specialists, a specialist nurse and patient support group leaders. Detailed outcome parameters assessed in the survey including sociodemographic factors, impact on working productivity and healthcare utilization is described in detail in a report by Martelletti et al. [16]

For the purposes of this study, information was included for age, gender, family and employment status. Migraine history, frequency and severity of attacks, medication use and reported comorbidities were included. Information on healthcare resource utilization based on reported number of migraine-related visits to health care practitioners (HCP) in the past 6 months as well as emergency room (ER) visits and inpatient days (IPD) in the past 12 months were also included. The impact of migraine on work productivity and daily activities among employed respondents was evaluated by using the Work Productivity and Activity Impairment (WPAI) questionnaire [23] and complemented with additional work-related questions. The impact of migraine on daily functioning and the migraine-related emotional burden was evaluated by responses relating to the experience of living with migraine as well as the impact on social life.

Before the analyses were carried out the participants were categorized into three groups: 4 ≤ MMD < 8, 8 ≤ MMD < 15 and MMD ≥ 15, according to the reported mean monthly migraine days (MMDs) (determined by the self-reported number of migraine days in the past 3 months and divided by 3).

### Data analysis

For the continuous and normally distributed variables, the difference in mean between the groups was tested using the one-way ANOVA. Normality was assessed using histograms and Shapiro-Wilk test (data not shown). If the normality assumption was violated, the non-parametric Kruskall-Wallis test was utilised instead. For the categorical variables, the difference in group sizes was tested using the Chi-squared test when all the group sizes were above 5. If at least one group size was ≤5 the Fisher’s exact test was utilised instead. If the number of rows in the contingence table became ≥7, Monte Carlo simulation with 2000 replicates was utilised to compute the *p*-values for the Fisher’s test. A significance level of 0.05 was assumed throughout the analysis.

When assessing the number of sick-leave days from work due to the migraine during the previous 1 month, the questionnaire option “I did not work at all during the previous one month” was set to equal to 23 missed workdays. When analysing questions related to the severity of pain, daily functioning and emotional burden, the questionnaire options were re-categorized into two levels instead of the original five. The options “a lot” or “often” and “always” were grouped as one level, while the rest, i.e. “no impact”, “slight impact”, and “moderate impact”, were grouped as another level.

The work productivity and activity impairment related to a specific healthcare problem [24] (WPAI-SHP) was evaluated only for the respondents who reported current employment (*n* = 227). The WPAI outcomes are expressed as percentages of impairment, as higher WPAI percentage indicates greater impairment and lower productivity.

The relationship between healthcare resource utilization and selected predictors, i.e. age, gender, MMDs and the number of comorbidities, was assessed using regression analysis. The dependent variables, i.e. the number of HCP visits, ER visits and inpatient days, were over-dispersed and therefore, the negative binomial regression analysis was performed.

All analyses were conducted using R (version 3.6.2) [25].

## Results

### Patient demographics and clinical characteristics

The total number of respondents was 345, of which 338 fulfilled the inclusion criteria. The number of respondents in subgroups according to the self-reported average number of migraine days experienced in the previous 3 months were 133 (39.3%) for 4 ≤ MMD < 8 and 139 (41.4%) for 8 ≤ MMD < 15 corresponding to EM, and 66 (19.5%) for MMD ≥ 15 corresponding to CM [5].

The demographics of the cohort and subgroups are shown in Table 1. The majority of the respondents lived in South and West Finland (78%). The mean age was 44 years, the majority were female (93%), had children (66%) and reported a family history of migraine (86%), with no significant difference between the subgroups. Seventy-six percent of the study participants reported migraine for 16 years or more. The respondents recruited via PAG (*n* = 257, 76%) reported having migraine significantly longer times as compared to those recruited via GfK Health (*N* = 81, 26%) (data shown in Additional file 1).

**Table 1:** Demographics among respondents stratified by mean monthly migraine days (MMDs)

	Overall	4 ≤ MMD < 8	8 ≤ MMD < 15	MMD ≥ 15	***p***-value
**N**	338	133	139	66	
**Age (mean (SD))**	43.6 (11.5)	43.4 (11.5)	43.4 (11.2)	44.6 (12.1)	0.724
**Mean headache days incl. Migraine (mean (SD))**	12.4 (6.1)	8.2 (4.2)	12.5 (4.0)	20.6 (4.4)	**< 0.001**
**Gender (%)**					
Female	314 (92.9)	123 (92.5)	132 (95.0)	59 (89.4)	0.339
Male	24 (7.1)	10 (7.5)	7 (5.0)	7 (10.6)	
**Employed**	227 (67.2)	92 (69.2)	97 (69.8)	38 (57.6)	0.180
**Time being affected by migraine (%)**					
0–5 years	16 (4.7)	5 (3.8)	7 (5.0)	4 (6.1)	0.366
6–15 years	73 (21.6)	36 (27.1)	26 (18.7)	11 (16.7)	
16 or more years	249 (73.7)	92 (69.2)	106 (76.3)	51 (77.3)	
**Comorbidities**					
Chronic pain (%)	79 (23.4)	22 (16.5)	36 (25.9)	21 (31.8)	**0.037**
Cardiometabolic disorders (%)	141 (41.7)	62 (46.6)	51 (36.7)	28 (42.4)	0.250
Mental health-related (%)	118 (34.9)	42 (31.6)	49 (35.3)	27 (40.9)	0.427
Other (%)	180 (53.3)	68 (51.1)	74 (53.2)	38 (57.6)	0.692
**Responsible HCP for treating migraine (%)**					
General practitioner	111 (32.8)	49 (36.8)	48 (34.5)	14 (21.2)	**0.007**
Neurologist	111 (32.8)	31 (23.3)	49 (35.3)	31 (47.0)	
Other	16 (4.7)	6 (4.5)	6 (4.3)	4 (6.1)	
Myself instead of any HCP	77 (22.8)	41 (30.8)	26 (18.7)	10 (15.2)	
No one	23 (6.8)	6 (4.5)	10 (7.2)	7 (10.6)	

Age and mean headache days are presented as mean +/− SD (standard deviation) and other variables as absolute number and percentage of respondents. Grouped comorbidities are presented in more detail in the Additional file 2

*HCP* healthcare practitioner

The mean number of reported comorbidities was 2.4. Comorbidities were reported to have been diagnosed after the first migraine attack in a majority of cases (Additional file 3). The most common comorbidities were allergies (34%), obesity (28%), chronic gastrointestinal diseases (23%), insomnia or sleep disorder (21%) and chronic pain (19%). No distribution difference between subgroups was observed.

The HCP responsible for treating migraine in the 4 ≤ MMD < 8 subgroup was most often a general practitioner (GP) (37%) and in the MMD ≥ 15 a neurologist (47%).

Reported migraine medications are shown in Table 2. Majority (94%) reported use of acute medication, which had mainly been prescribed by their treating physician (98%) and complemented by over-the-counter medication (OTC) (43%). Triptans and pain relievers were most commonly used (81% and 80%, respectively) and the use was similar in all subgroups. Thirty percent of the respondents reported using complementary non-pharmaceutical therapies.

**Table 2:** Use of treatments among respondents overall and stratified by mean monthly migraine days (MMDs)

	Overall	4 ≤ MMD < 8	8 ≤ MMD < 15	MMD ≥ 15	***p***-value
**N**	338	133	139	66	
**Use of acute treatment (%)**	319 (94.4)	128 (96.2)	129 (92.8)	62 (93.9)	0.455
**Currently used acute medication (%)**					
Non-opioid pain relievers	254 (80.4)	101 (78.9)	109 (85.8)	44 (72.1)	0.074
Triptans	256 (81.0)	98 (76.6)	106 (83.5)	52 (85.2)	0.240
Anti-emetics	112 (35.4)	41 (32.0)	43 (33.9)	28 (45.9)	0.157
Any other	70 (22.2)	23 (18.0)	25 (19.7)	22 (36.1)	**0.014**
**Currently used acute treatment (%)**					
Medicine prescribed by a doctor	311 (97.5)	126 (98.4)	125 (96.9)	60 (96.8)	0.717
OTC medicine	138 (43.3)	55 (43.0)	60 (46.5)	23 (37.1)	0.468
Non-medical complementary therapies*	95 (29.8)	37 (28.9)	41 (31.8)	17 (27.4)	0.795
Any other	45 (14.1)	15 (11.7)	22 (17.1)	8 (12.9)	0.449
**Ever received prophylactic prescription (%)**	281 (83.1)	103 (77.4)	116 (83.5)	62 (93.9)	**0.010**
**Currently used prophylactic medications (%)**					
Beta-blockers	121 (43.1)	49 (47.6)	49 (42.2)	23 (37.1)	0.409
Anti-epileptics	97 (34.5)	29 (28.2)	47 (40.5)	21 (33.9)	0.157
Anti-depressants	56 (19.9)	17 (16.5)	24 (20.7)	15 (24.2)	0.471
Onabotulinum toxin A	43 (15.3)	4 (3.9)	17 (14.7)	22 (35.5)	**< 0.001**
Any other	78 (27.8)	29 (28.2)	32 (27.6)	17 (27.4)	0.993
**Number of prophylactic treatment switches/failures (%)**					
Never	63 (22.4)	37 (35.9)	18 (15.5)	8 (12.9)	**< 0.001**
Once	21 (7.5)	12 (11.7)	5 (4.3)	4 (6.5)	
Twice	29 (10.3)	15 (14.6)	10 (8.6)	4 (6.5)	
3 times	47 (16.7)	13 (12.6)	29 (25.0)	5 (8.1)	
4 times	25 (8.9)	8 (7.8)	9 (7.8)	8 (12.9)	
5 times	20 (7.1)	4 (3.9)	11 (9.5)	5 (8.1)	
6 times or more	76 (27.0)	14 (13.6)	34 (29.3)	28 (45.2)	

*Not specified

All variables are presented as absolute number and percentage of respondents

*OTC* over-the-counter

Prophylactic treatment typically included only one medication (Additional file 4) whereas attack treatment was most often a combination of two or three medications, and use of different combinations differed significantly between MMD groups. Majority of cases (69%) were totally or somewhat satisfied with their current acute treatment in all subgroups (Additional file 5). Minority of respondents were able to define the duration of use of their current acute medication.

Prophylactic medications used in Finland included oral medication and injectable onabotulinum toxin A (indication in CM), excluding candesartan (off-label) [26]. A total of 83% reported at least one prescription for prophylactic medication during their migraine history and 77.6% of them reported one to six medication switches. The number of prophylactic medications was highest in MMD ≥15 group (94%, *p* = 0.01) where up to 45% had switched the medication 6 times or more. In this cohort onabotulinum toxin A was most commonly used in MMD ≥ 15 group and the observed use in other MMD subgroups may indicate a transformation of CM to EM over the treatment period.

General satisfaction to the current prophylactic medication showed different distribution in subgroups and was lowest in MDD ≥ 15 group (Additional file 5). Sixteen questions evaluated the satisfaction for prophylactic medication, reported in 37.0% (N 125/338). Users in all subgroups reported improved control over migraines in total of 39.2% and improved quality of life in 48.8%.

Dissatisfaction in current prophylactic medications was evaluated in nine questions, reported in 25.9% (N 87/338). The causes concerned mainly the lack of efficacy (58.6%) and side-effects (44.8%), which results were similar in all subgroups.

### Impact on severity of pain, daily functioning and emotional burden

The negative impact of migraine on daily functioning and the emotional burden of migraine over the previous 1 month are described in Table 3. In all subgroups the mean severity of pain was high, 7.5–7.8, assessed by pain severity scale from zero to 10. Majority of cases (75%) reported spending long periods of time in darkness or isolation.

**Table 3:** Daily functioning and emotional burden in migraine

Impact	Overall	4 ≤ MMD < 8	8 ≤ MMD < 15	MMD ≥ 15	***p***-value
N	338	133	139	66	
**Symptom burden**					
Severity of pain (range from 0 to 10; mean (SD))	7.6 (1.5)	7.5 (1.5)	7.6 (1.5)	7.8 (1.3)	0.570
Ever spent long periods in darkness or isolated (%)	253 (74.9)	94 (70.7)	105 (75.5)	54 (81.8)	0.227
Migraine decreases the quality of life (“a lot” or “significantly”; %)	198 (58.6)	52 (39.1)	97 (69.8)	49 (74.2)	**< 0.001**
**Emotional burden**					
Often or always feeling frustrated by migraine (%)	200 (59.2)	57 (42.9)	99 (71.2)	44 (66.7)	**< 0.001**
Often or always feeling hopeless or helpless by migraine (%)	123 (36.4)	37 (27.8)	54 (38.8)	32 (48.5)	**0.013**
Level of fear of migraine attacks (“a lot” or “significantly”; %)	74 (21.9)	18 (13.5)	41 (29.5)	15 (22.7)	**0.006**
**Daily functioning**					
Ever cancelled plans due to migraine (%)	317 (93.8)	116 (87.2)	136 (97.8)	65 (98.5)	**< 0.001**
Migraine often or always interfering with ability to think clearly or to focus on daily life activities and tasks (%)	211 (62.4)	59 (44.4)	99 (71.2)	53 (80.3)	**< 0.001**
Level of migraine interference with daily activities (“a lot” or “constantly”; %)	206 (60.9)	56 (42.1)	97 (69.8)	53 (80.3)	**< 0.001**
Level of impairment in daily activity due to migraine (needing to stop and rest “a lot” or “always”; %)	220 (65.1)	67 (50.4)	100 (71.9)	53 (80.3)	**< 0.001**
Often or always lacking the energy to complete daily living or felt fatigued (%)	137 (40.5)	38 (28.6)	60 (43.2)	39 (59.1)	**< 0.001**
Ever had troubles of sleeping (%)	250 (74.0)	91 (68.4)	102 (73.4)	57 (86.4)	**0.025**

The impact of migraine on daily functioning and emotional reactions among respondents in subgroups by mean monthly migraine days (MMDs). Results are presented as absolute number and percentage of respondents

Significant impairment was observed in quality of life domains, resulting in a negative impact of migraine on daily functioning and emotional reactions. Overall, more than 60% of the respondents reported that they had to cancel plans due to migraine and that migraine interfered with daily activities and ability to think clearly. Trouble sleeping, thoughts of hopelessness, helplessness or frustration and fear of migraine attacks were frequently reported. Both emotional reactions and problems in daily functioning showed different distribution in subgroups, difference was significant.

### Impact on working life

Sixty-seven percent (*n* = 227) of the respondents were fully or part-time employed or entrepreneurs. A high proportion (90%) reported that their current employer is aware of the migraine, whereas only 46% of the employers offered support to the migraine patients.

**Fig. 1:**
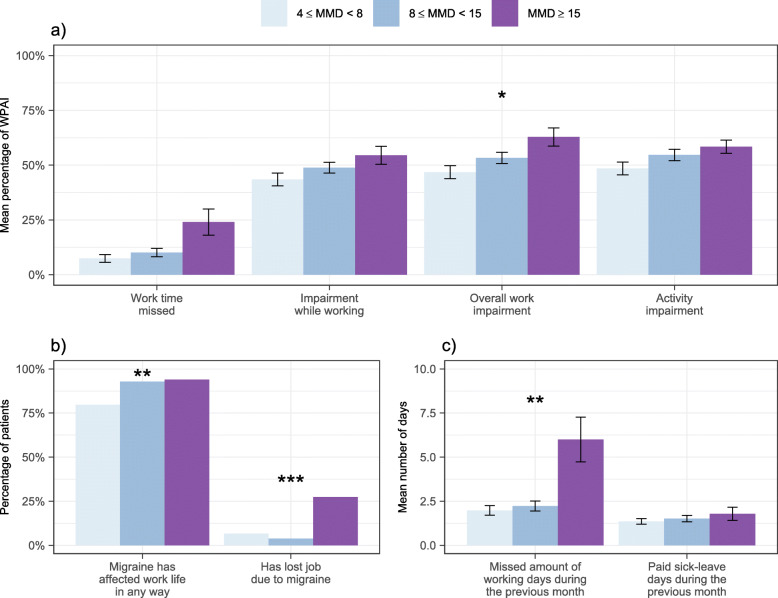
The impact of migraine on work productivity and activity impairment (WPAI) during previous 1 week (**a**), overall working life (**b**) and missed amount of working days and paid sick-leave days (**c**) during the previous 1 month stratified by mean monthly migraine days (MMDs). WPAI was evaluated only for those respondents, who reported being currently working (*N* = 227). The height of the bar indicates the mean while the accompanying whiskers in figures **a** and **c** represent the standard errors (SE). *p < 0.05, **p < 0.01, ***p < 0.001

Eighty-eight percent of the survey participants reported that migraine had affected their overall work life (Fig. 1b). The rate differed significantly between the subgroups (*p* = 0.001). Mainly individuals in MMD ≥ 15 group reported having lost their job due to migraine (27%, *p* < 0.001).

More than one working day was missed due to migraine over the previous 1 month, the overall mean being 2.8 days, of which 1.5 (54%) were paid sick leave days (Fig. 1c). In MMD ≥ 15 group the average number of missed working days due to the migraine was higher, 6.0 days, and the number of paid sick leave days was only 1.8 days (31%).

Over the past 7 days migraine caused a reduction in working time (absenteeism) in 11% and a reduction in impairment while working (presenteeism) in 48% and the corresponding rates in the MMD ≥ 15 group were 24% and 55%, respectively (Fig. 1a). The overall work productivity impairment differed significantly between the subgroups being highest in the MMD ≥ 15 group (4 ≤ MMD < 8: 47%; 8 ≤ MMD < 15: 53%; and MMD ≥ 15: 63% (*p* = 0.01)).

Fifty-three percent of the survey participants reported impairment in daily activities including homework, shopping and hobbies, and the outcome tended to differ between the subgroups (*p* = 0.06).

### Health care resource utilization

**Fig. 2:**
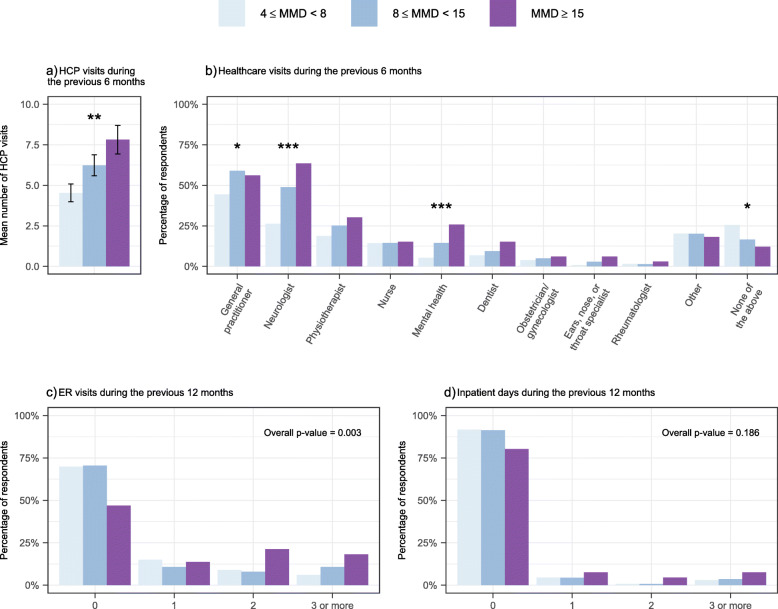
Mean number of healthcare practitioner (HCP) visits during the previous 6 months (**a**; mean and SE), the percentage of reported healthcare visits (**b**), visits to emergency room (ER) (**c**) and reported inpatient days during the previous 12 months (**d**) stratified by mean monthly migraine days (MMDs). *p < 0.05, **p < 0.01, ***p < 0.001

During the previous 6 months 81% reported at least one migraine-related visit at healthcare practitioner (HCP), the mean being 5.9 visits (Fig. 2a). The number of HCP visits was higher in the MMD ≥ 15 group compared to the 4 ≤ MMD < 8 group (*p* = 0.001), Fig. 2a. The subgroups reported significantly different rates of visits at general practitioner, neurologist, and mental health professionals, including psychologist and psychiatrist (*p* < 0.05, *p* < 0.001, and *p* < 0.001, respectively, Fig. 2b). Overall, the survey participants visited most often a GP or a neurologist (Fig. 2b).

During the previous 12 months, 34% reported at least one ER visit. There was a trend towards a higher number of ER visits with increasing migraine frequency (Fig. 2c). Eleven percent reported having at least one inpatient day and there was no difference between the groups (Fig. 2d).

**Fig. 3:**
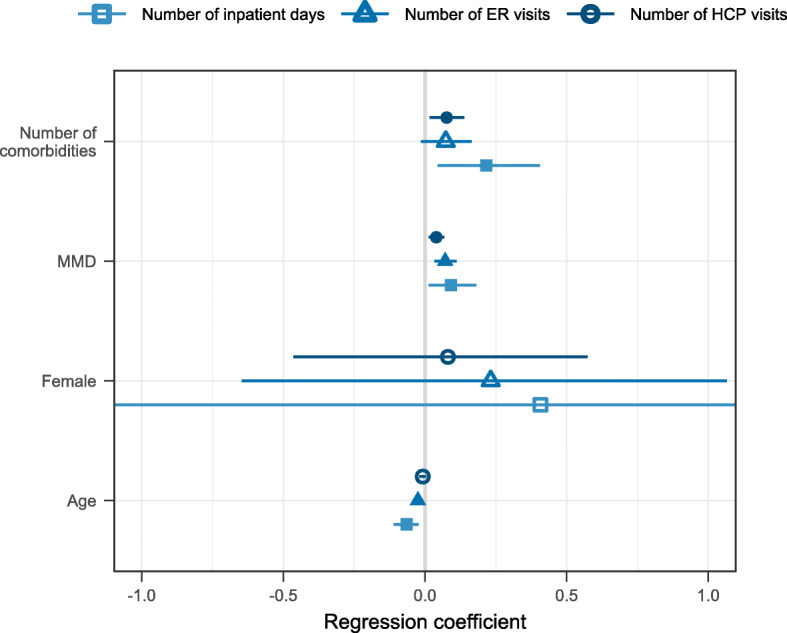
Forest plot of the regression results of analysing the number of HCP visits during the previous 6 months, and the number of ER visits as well as inpatient days during the previous 12 months. The shapes indicate the regression coefficient estimate and the accompanying line indicates the 95% CI. A filled shape indicates a significant association (*p* < 0.05). The x-axis is in the natural logarithmic scale meaning that 0.08 increase in the dependent variable (e.g. number of comorbidities for the number of HCP visits) means 1.08 actual increase, i.e. 8% increase. This increase is reflected by one unit increase in the independent variable, meaning e.g. one more comorbidity or one more monthly migraine day. Exact values of coefficients, exponentiated coefficients, CIs, and *p*-values are presented in Additional file 6

The association of the HRCU, i.e. the number of HCP visits during the previous 6 months and the number of ER visits and inpatient days during the previous 12 months with age, gender, number of comorbidities, and MMDs, was assessed using multivariable negative binomial regression. The effect of each variable on the healthcare resource utilization was consistent throughout the HCRU types even though the impact did not reach statistical significance for all the variables (Fig. 3). A higher number of any comorbidity was associated with a higher migraine related HCRU. Also, higher number of MMDs was associated with increased HCRU with inpatient days appearing the most affected. However, the CIs are generally wider for the regression coefficients for the inpatient days as only 11% of individuals had at least one inpatient day during the previous 12 months. Note that the MMD was included as a continuous variable in the regression analysis.

## Discussion

Our main results drawn from the Finnish subset of global *My Migraine Voice* survey data showed a tendency towards worse outcomes in a wide set of domains assessing migraine burden, consistent with increase in migraine frequency detected by MMDs. The results are consistent with observations in recently published surveys in European countries [27] and in Sweden [20]. These studies, the global *My Migraine Voice* survey and other observations [13, 15, 28] indicate that individuals with episodic or chronic migraine report worse health status and negative impact on activities and working life.

The current data from Finland has not been reported previously. Majority of cases in the Finnish high frequency migraine cohort were working age and women. In the total cohort, the employment rate (67%) corresponded to the European female employment rate in 2018 [29]. It has been estimated that a majority, 77% to 93%, of all costs associated with overall migraine population are indirect and attributed to impaired or lost work productivity [20, 30, 31]. In our data, the common WPAI domains assessing indirectly the economic burden of migraine showed that over half of the respondents reported overall work impairment (absenteeism and presenteeism), and the results corroborate the reported loss in overall work productivity in other studies [11–13, 15]. Loss of productivity was determined by the total percentage of missed time at work, which was higher than the rate reported in the European EU5 study [27]. The increase in the mean number of missed workdays was 3-fold in CM (6 days) as compared to the other subgroups and the rates for absenteeism and presenteeism together differed between the subgroups (Fig. 1a). Furthermore, employment in CM (58%) was lower and the rate of lost jobs (27%) higher as compared to total in other groups.

The health care resource utilization and economic impact to the healthcare system increases along with the frequency of migraine days and the severity of migraine. Our aim was to characterize more precisely the association of migraine frequency with HCRU and facilitate the interpretation of the regression coefficients. Our results indicate that a one-day increase of monthly migraine days reflects an increase of 4%, 7% and 10% in HCP visits, ER visits and inpatient days, respectively. More precisely, an increase in the number of mean monthly migraine days e.g. from 5 to 20 days, equivalent to progression from the 4 ≤ MMD < 8 group to the MMD ≥ 15 group, indicates an increase of approximately 80%, 190% and 300% in HCP visits, ER visits, and inpatient days, respectively. Our results corroborate other observations and a high need of versatile HCP consultations, which increased markedly with migraine frequency. The rate of respondents reporting neurologist visits varied from 26% in 4 ≤ MMD < 8 up to 64% in MMD ≥ 15 group. The number of visits at GP and neurologist were frequent and the reported rates were higher than reported in European EU5 study [27] and in other European studies [32, 33]. Difference may be explained by differences in health care system and insurance practice.

The direct costs of migraine care increase further by the presence of comorbidities [12, 34, 35]. Our results on increasing HCP visits, inpatient days and ER visits by both severity of migraine and comorbidities are consistent with these studies. Mental health related issues in migraine are frequent and lifetime prevalence of depression is high, reports varying from 18.8 to 42% [36–38]. The reported mental health issues in our data, most often insomnia, anxiety and depression, were reported in 35% of the total cohort and in 41% of those individuals with CM. The rate was higher than for psychiatric diseases (22%) in a Swedish study, however the direct comparisons between studies are hampered due to use of different inclusion of the reported diseases. A study among Finnish migraine families demonstrated that especially women were likely to have additional disorders, such as hypotension, allergies and psychiatric disorders besides their migraine [18]. Other comorbidities frequently related to migraine are painful musculoskeletal disorders reported in a Finnish study among working aged suffering from migraine [39]. Among the Finnish *My Migraine Voice* survey participants rheumatoid arthritis, chronic joint inflammation, fibromyalgia and chronic back pain were reported in almost one fifth (17.8%) of all cases. This finding is in line with previous studies that have shown that migraine is highly comorbid with other chronic pain syndromes [40]. Migraine and other chronic pain syndromes have shown to enhance pain and hyperalgesia in patients with fibromyalgia [40, 41]. Cardiometabolic conditions along with several other conditions were also frequent. Increasing number of any of the reported comorbidities increased the HCP visits by 8%, ER visits by 8%, and inpatient days by 24%. These results likely reflect the two-way street of comorbidity related to frequent migraine in adult population.

The burden of migraine in *My Migraine Voice* survey covered several domains of the individual’s personal life and emotional responses to migraine. The results point to that the majority of the respondents experience severe pain and need for isolation during attacks. Also the general negative impact on QoL increased significantly by MMD frequency. The same was true for emotional burden, including reported fear, frustration, or hopelessness. The burden of migraine is shown to relate to decreased functional ability during ictal and interictal phases [30, 31, 42, 43] indicating that the reported emotional responses and the reported mental comorbidities are closely linked.

The majority of the individuals in this dataset were treated by a physician, which likely explains the high use of triptans in 81% and prophylactic medications in 83%. These prescription patterns are considerably higher than previously reported among Finnish working-aged migraineurs [44] or in the Swedish and AMPP studies [45, 46]. Chronic migraine, failed prophylactic treatments, and several switches characterize the difficult-to-treat migraine population also in our data. Onabotulintoxin A has indication and reimbursement for CM in Finland, and the use of it was observed in all subgroups in our data, however less than that reported in a Swedish study [46]. Our results points to a transition from chronic to episodic migraine among users of onabotulinum toxin, indicated in chronic migraine. The results emphasize the need for other treatment options when traditional oral treatments fail. Today other treatments include also CGRP monoclonal antibodies [47]. The treatment escalation paradigm is supported by our observation that migraine frequency was associated with a significant change in a wide range of study parameters addressing the burden of migraine.

*My Migraine Voice* was not designed to be population-based and the design was cross-sectional, and thus no follow-up data is available. Our Finnish subsample represents a small proportion of the mainly female migraine-sufferers. Also, all data in the *My Migraine Voice* survey was patient-reported via an online panel-based sample and there are several limitations which are inherent to these type of studies [48, 49]. Selection bias and nonresponse bias cannot be estimated in this study. Another limitation is the use of self-reported data. Self-reporting of migraine has been used in several studies and is considered reliable, supported here by a high number of respondents from PAG [50]. Also, major HCP events, like ER visits or hospitalizations, are less likely to be subjected to inaccurate recall compared to a visit to the general practitioner, however these visits or other variables of interest were not clinically confirmed. Certain other biases may exist; therefore, caution needs to be taken when interpreting the data. However, we assume that our findings can be generalised to most individuals having either episodic or chronic migraine.

## Conclusion

We have here presented the results from the global *My Migraine Voice survey* in the Finnish sample and shown that the disease burden increases according to migraine severity. The cohort characterized a population where migraine exhibited the strongest effect both on working and personal life. Increase in migraine frequency was associated with greater productivity loss and use of healthcare resources as well as the effects conveyed to personal and societal level. The results point to the need to lessen this burden and to consider more active use of effective prophylactic treatments.

## Supplementary information

**Additional file 1.** Prophylactic medication and time being affected by migraine stratified by recruitment path (GfK Health/ Patient Advocacy Group (PAG)). Results are presented as absolute number and percentage of respondents.

**Additional file 2.** Comorbidities of the study respondents overall and stratified by mean monthly migraine days (MMDs). Results are presented as absolute number and percentage of respondents.

**Additional file 3.** Timing of reported comorbidities in relation to the first migraine attack (before/after).

**Additional file 4.** Duration using each type of acute and prophylactic medication and their combinations.

**Additional file 5.** Satisfaction and dissatisfaction in current acute and prophylactic medication and specific causes in these groups (prophylactic medication).

**Additional file 6.** Results from the negative binomial regression analysis.

## Data Availability

The datasets generated and/or analyzed during the current study are not publicly available for data confidentiality reasons.

## Abbreviations

MMD: Monthly migraine days
WPAI: Work productivity and activity impairment
HCRU: Healthcare resource utilization
HCP: Healthcare practitioner
ER: Emergency room
CM: Chronic migraine
EM: Episodic migraine
QoL: Quality of life
PAG: Patient advocacy group
OBB: Online bulletin board
IPD: Inpatient day
OTC: Over the counter
