# Supplementary material for: My Migraine Voice survey: disease impact on healthcare resource utilization, personal and working life in Finland
Source: J Headache Pain. 2020 Sep 29;21(1):118. doi: 10.1186/s10194-020-01185-4 (PMC7526198; doi:10.1186/s10194-020-01185-4)
Supplement: Supplementary file 1 — Additional file 1. Prophylactic medication and time being affected by migraine stratified by recruitment path (GfK Health/ Patient Advocacy Group (PAG)). Results are presented as absolute number and percentage of respondents. [file 10194_2020_1185_MOESM1_ESM.docx]

**Additional file 1.** Prophylactic medication and time being affected by migraine stratified by recruitment path (GfK Health/ Patient Advocacy Group (PAG)).

|  | **Overall** | **GfK Health** | **PAG** | **p-value** |
| --- | --- | --- | --- | --- |
| N | 338 | 81 | 257 |  |
| **Ever received prophylactic prescription (%)** | 281 (83.1) | 60 (74.1) | 221 (86.0) | **0.020** |
| **Time being affected by migraine (%)** | | | | **<0.001** |
| 0-5 years | 16 (4.7) | 9 (11.1) | 7 (2.7) |  |
| 6-15 years | 73 (21.6) | 26 (32.1) | 47 (18.3) |  |
| 16 or more years | 249 (73.7) | 46 (56.8) | 203 (79.0) |  |

Results are presented as absolute number and percentage of respondents.
