# Supplementary material for: My Migraine Voice survey: disease impact on healthcare resource utilization, personal and working life in Finland
Source: J Headache Pain. 2020 Sep 29;21(1):118. doi: 10.1186/s10194-020-01185-4 (PMC7526198; doi:10.1186/s10194-020-01185-4)
Supplement: Supplementary file 2 — Additional file 2. Comorbidities of the study respondents overall and stratified by mean monthly migraine days (MMDs). Results are presented as absolute number and percentage of respondents. [file 10194_2020_1185_MOESM2_ESM.docx]

**Additional file 2.** Comorbidities of the study respondents overall and stratified by mean monthly migraine days (MMDs).

| **Comorbidities** | **Overall** | **4 ≤ MMD < 8** | **8 ≤ MMD < 15** | **MMD ≥ 15** | **p-value** |
| --- | --- | --- | --- | --- | --- |
| N | 338 | 133 | 139 | 66 |  |
| **Chronic pain** | | | | | |
| Chronic pain (%) | 63 (18.6) | 16 (12.0) | 29 (20.9) | 18 (27.3) | **0.023** |
| Chronic back pain (%) osteoarthritis | 33 (9.8) | 9 (6.8) | 14 (10.1) | 10 (15.2) | 0.170 |
| **Cardiometabolic disorders** | | | | | |
| Diabetes type 1 (%) | 3 (0.9) | 1 (0.8) | 0 (0.0) | 2 (3.0) | 0.052 |
| Diabetes type 2 (%) | 12 (3.6) | 8 (6.0) | 1 (0.7) | 3 (4.5) | **0.039** |
| Obesity (%) | 93 (27.5) | 41 (30.8) | 38 (27.3) | 14 (21.2) | 0.359 |
| Cardiovascular disease (%) | 20 (5.9) | 11 (8.3) | 6 (4.3) | 3 (4.5) | 0.373 |
| High blood pressure (%) | 56 (16.6) | 26 (19.5) | 18 (12.9) | 12 (18.2) | 0.317 |
| High cholesterol (%) | 28 (8.3) | 13 (9.8) | 7 (5.0) | 8 (12.1) | 0.165 |
| **Mental health-related** | | | | | |
| Anxiety (%) | 49 (14.5) | 19 (14.3) | 19 (13.7) | 11 (16.7) | 0.847 |
| Depression (%) | 58 (17.2) | 26 (19.5) | 19 (13.7) | 13 (19.7) | 0.363 |
| Insomnia/sleep disorder (%) | 70 (20.7) | 25 (18.8) | 28 (20.1) | 17 (25.8) | 0.510 |
| Chronic fatigue syndrome (%) | 10 (3.0) | 2 (1.5) | 5 (3.6) | 3 (4.5) | 0.407 |
| **Other disorders** | | | | | |
| Asthma (%) | 37 (10.9) | 13 (9.8) | 15 (10.8) | 9 (13.6) | 0.712 |
| Epilepsy (%) | 4 (1.2) | 0 (0.0) | 3 (2.2) | 1 (1.5) | 0.251 |
| Allergy (%) | 113 (33.4) | 39 (29.3) | 47 (33.8) | 27 (40.9) | 0.262 |
| Rheumatoid arthritis/chronic joint inflammation/fibromyalgia (%) | 36 (10.7) | 13 (9.8) | 15 (10.8) | 8 (12.1) | 0.878 |
| Osteoporosis (%) | 6 (1.8) | 2 (1.5) | 2 (1.4) | 2 (3.0) | 0.749 |
| Chronic gastrointestinal diseases (%) | 77 (22.8) | 27 (20.3) | 34 (24.5) | 16 (24.2) | 0.681 |
| Other (not specified) (%) | 58 (17.2) | 18 (13.5) | 27 (19.4) | 13 (19.7) | 0.362 |
| **None of the above (%)** | 72 (21.3) | 31 (23.3) | 28 (20.1) | 13 (19.7) | 0.766 |

Results are presented as absolute number and percentage of respondents.
