# Supplementary material for: My Migraine Voice survey: disease impact on healthcare resource utilization, personal and working life in Finland
Source: J Headache Pain. 2020 Sep 29;21(1):118. doi: 10.1186/s10194-020-01185-4 (PMC7526198; doi:10.1186/s10194-020-01185-4)
Supplement: Supplementary file 3 — Additional file 3. Timing of reported comorbidities in relation to the first migraine attack (before/after). [file 10194_2020_1185_MOESM3_ESM.docx]

**Additional file 3.** Timing of reported comorbidities in relation to the first migraine attack (before/after).

|  |  | **Overall** | **4 ≤ MMD < 8** | **8 ≤ MMD < 15** | **MMD ≥ 15** | **p-value** |
| --- | --- | --- | --- | --- | --- | --- |
| **Chronic pain** | | | | | | |
| Chronic pain, N (%)  Total 63/338 | Before my first migraine attack | 6 (9.5) | 0 (0.0) | 4 (13.8) | 2 (11.1) | 0.496 |
|  | After my first migraine attack | 49 (77.8) | 15 (93.8) | 20 (69.0) | 14 (77.8) |  |
|  | Not able to define | 8 (12.7) | 1 (6.2) | 5 (17.2) | 2 (11.1) |  |
| Chronic back pain, N (%)  Total 33/338 | Before my first migraine attack | 7 (21.2) | 0 (0.0) | 4 (28.6) | 3 (30.0) | 0.137 |
|  | After my first migraine attack | 23 (69.7) | 9 (100.0) | 9 (64.3) | 5 (50.0) |  |
|  | Not able to define | 3 (9.1) | 0 (0.0) | 1 (7.1) | 2 (20.0) |  |
| **Cardiometabolic disorders** | | | | | | |
| Type 1 diabetes, N (%)  Total 3/338 | Before my first migraine attack | 3 (100.0) | 1 (100.0) | 0 (-) | 2 (100.0) | 1.000 |
|  | After my first migraine attack | 0 (0.0) | 0 (0.0) | 0 (-) | 0 (0.0) |  |
|  | Not able to define | 0 (0.0) | 0 (0.0) | 0 (-) | 0 (0.0) |  |
| Type 2 diabetes, N (%)  Total 12/338 | Before my first migraine attack | 2 (16.7) | 2 (25.0) | 0 (0.0) | 0 (0.0) | 1.000 |
|  | After my first migraine attack | 9 (75.0) | 5 (62.5) | 1 (100.0) | 3 (100.0) |  |
|  | Not able to define | 1 (8.3) | 1 (12.5) | 0 (0.0) | 0 (0.0) |  |
| Obesity, N (%)  Total 93/338 | Before my first migraine attack | 13 (14.0) | 5 (12.2) | 8 (21.1) | 0 (0.0) | 0.112 |
|  | After my first migraine attack | 69 (74.2) | 33 (80.5) | 26 (68.4) | 10 (71.4) |  |
|  | Not able to define | 11 (11.8) | 3 (7.3) | 4 (10.5) | 4 (28.6) |  |
| Cardiovascular disease,  N (%)  Total 20/338 | Before my first migraine attack | 5 (25.0) | 2 (18.2) | 2 (33.3) | 1 (33.3) | 0.221 |
|  | After my first migraine attack | 13 (65.0) | 9 (81.8) | 3 (50.0) | 1 (33.3) |  |
|  | Not able to define | 2 (10.0) | 0 (0.0) | 1 (16.7) | 1 (33.3) |  |
| High blood pressure,  N (%)  Total 56/338 | Before my first migraine attack | 8 (14.3) | 4 (15.4) | 0 (0.0) | 4 (33.3) | 0.066 |
|  | After my first migraine attack | 42 (75.0) | 18 (69.2) | 16 (88.9) | 8 (66.7) |  |
|  | Not able to define | 6 (10.7) | 4 (15.4) | 2 (11.1) | 0 (0.0) |  |
| High cholesterol, N (%)  Total 28/338 | Before my first migraine attack | 4 (14.3) | 2 (15.4) | 1 (14.3) | 1 (12.5) | 0.822 |
|  | After my first migraine attack | 21 (75.0) | 10 (76.9) | 6 (85.7) | 5 (62.5) |  |
|  | Not able to define | 3 (10.7) | 1 (7.7) | 0 (0.0) | 2 (25.0) |  |
| **Mental health related** | | | | | | |
| Anxiety, N (%)  Total 49/338 | Before my first migraine attack | 8 (16.3) | 3 (15.8) | 3 (15.8) | 2 (18.2) | 1.000 |
|  | After my first migraine attack | 37 (75.5) | 15 (78.9) | 14 (73.7) | 8 (72.7) |  |
|  | Not able to define | 4 (8.2) | 1 (5.3) | 2 (10.5) | 1 (9.1) |  |
| Depression, N (%)  Total 58/338 | Before my first migraine attack | 12 (20.7) | 6 (23.1) | 5 (26.3) | 1 (7.7) | 0.575 |
|  | After my first migraine attack | 40 (69.0) | 18 (69.2) | 11 (57.9) | 11 (84.6) |  |
|  | Not able to define | 6 (10.3) | 2 (7.7) | 3 (15.8) | 1 (7.7) |  |
| Insomnia/sleep disorder,  N (%)  Total 70/338 | Before my first migraine attack | 11 (15.7) | 4 (16.0) | 6 (21.4) | 1 (5.9) | 0.578 |
|  | After my first migraine attack | 45 (64.3) | 17 (68.0) | 15 (53.6) | 13 (76.5) |  |
|  | Not able to define | 14 (20.0) | 4 (16.0) | 7 (25.0) | 3 (17.6) |  |
| Chronic fatigue syndrome, N (%)  Total 10/338 | Before my first migraine attack | 1 (10.0) | 0 (0.0) | 1 (20.0) | 0 (0.0) | 1.000 |
|  | After my first migraine attack | 9 (90.0) | 2 (100.0) | 4 (80.0) | 3 (100.0) |  |
|  | Not able to define | 0 (0.0) | 0 (0.0) | 0 (0.0) | 0 (0.0) |  |
| **Other disorders** | | | | | | |
| Asthma, N (%)  Total 37/338 | Before my first migraine attack | 6 (16.2) | 1 (7.7) | 3 (20.0) | 2 (22.2) | 0.681 |
|  | After my first migraine attack | 26 (70.3) | 10 (76.9) | 11 (73.3) | 5 (55.6) |  |
|  | Not able to define | 5 (13.5) | 2 (15.4) | 1 (6.7) | 2 (22.2) |  |
| Epilepsy, N (%)  Total 4/338 | Before my first migraine attack | 1 (25.0) | 0 (-) | 0 (0.0) | 1 (100.0) | 0.250 |
|  | After my first migraine attack | 3 (75.0) | 0 (-) | 3 (100.0) | 0 (0.0) |  |
|  | Not able to define | 0 (0.0) | 0 (-) | 0 (0.0) | 0 (0.0) |  |
| Allergy, N (%)  Total 113/338 | Before my first migraine attack | 53 (46.9) | 14 (35.9) | 26 (55.3) | 13 (48.1) | 0.189 |
|  | After my first migraine attack | 45 (39.8) | 16 (41.0) | 18 (38.3) | 11 (40.7) |  |
|  | Not able to define | 15 (13.3) | 9 (23.1) | 3 (6.4) | 3 (11.1) |  |
| Rheumatoid arthritis/chronic joint inflammation/ fibromyalgia, N (%)  Total 36/338 | Before my first migraine attack | 7 (19.4) | 2 (15.4) | 3 (20.0) | 2 (25.0) | 0.894 |
|  | After my first migraine attack | 25 (69.4) | 9 (69.2) | 10 (66.7) | 6 (75.0) |  |
|  | Not able to define | 4 (11.1) | 2 (15.4) | 2 (13.3) | 0 (0.0) |  |
| Osteoporosis, N (%)  Total 6/338 | Before my first migraine attack | 1 (16.7) | 0 (0.0) | 1 (50.0) | 0 (0.0) | 1.000 |
|  | After my first migraine attack | 5 (83.3) | 2 (100.0) | 1 (50.0) | 2 (100.0) |  |
|  | Not able to define | 0 (0.0) | 0 (0.0) | 0 (0.0) | 0 (0.0) |  |
| Chronic gastrointestinal disease, N (%)  Total 77/338 | Before my first migraine attack | 7 (9.1) | 3 (11.1) | 2 (5.9) | 2 (12.5) | 0.872 |
|  | After my first migraine attack | 63 (81.8) | 22 (81.5) | 28 (82.4) | 13 (81.2) |  |
|  | Not able to define | 7 (9.1) | 2 (7.4) | 4 (11.8) | 1 (6.2) |  |
| Other, N (%)  Total 58/338 | Before my first migraine attack | 12 (20.7) | 4 (22.2) | 6 (22.2) | 2 (15.4) | 0.581 |
|  | After my first migraine attack | 37 (63.8) | 10 (55.6) | 19 (70.4) | 8 (61.5) |  |
|  | Not able to define | 9 (15.5) | 4 (22.2) | 2 (7.4) | 3 (23.1) |  |
