# Supplementary material for: My Migraine Voice survey: disease impact on healthcare resource utilization, personal and working life in Finland
Source: J Headache Pain. 2020 Sep 29;21(1):118. doi: 10.1186/s10194-020-01185-4 (PMC7526198; doi:10.1186/s10194-020-01185-4)
Supplement: Supplementary file 4 — Additional file 4. Duration using each type of acute and prophylactic medication and their combinations. [file 10194_2020_1185_MOESM4_ESM.docx]

**Additional file 4.** Duration using each type of acute and prophylactic medication and their combinations.

|  | | **Overall** | **4 ≤ MMD < 8** | **8 ≤ MMD < 15** | **MMD ≥ 15** | **p-value** |
| --- | --- | --- | --- | --- | --- | --- |
| **Currently used acute medication: duration of use** | | | | | | |
| **Non opioid pain relievers, N (%)**  Total 319/338 | Less than a month | 2 (0.8) | 1 (1.0) | 1 (0.9) | 0 (0.0) | 0.922 |
|  | 1-6 months | 5 (2.0) | 4 (4.0) | 1 (0.9) | 0 (0.0) |  |
|  | 7-12 months | 20 (7.9) | 8 (7.9) | 9 (8.3) | 3 (6.8) |  |
|  | More than a year | 9 (3.5) | 3 (3.0) | 4 (3.7) | 2 (4.5) |  |
|  | Not able to define | 218 (85.8) | 85 (84.2) | 94 (86.2) | 39 (88.6) |  |
| **Triptans, N (%)**  Total 256/338 | Less than a month 1 | 2 (0.8) | 0 (0.0) | 1 (0.9) | 1 (1.9) | 0.721 |
|  | 1-6 months | 10 (3.9) | 2 (2.0) | 5 (4.7) | 3 (5.8) |  |
|  | 7-12 months | 19 (7.4) | 8 (8.2) | 9 (8.5) | 2 (3.8) |  |
|  | More than a year | 15 (5.9) | 6 (6.1) | 5 (4.7) | 4 (7.7) |  |
|  | Not able to define | 210 (82.0) | 82 (83.7) | 86 (81.1) | 42 (80.8) |  |
| **Anti-emetics, N (%)**  Total 112/338 | Less than a month | 3 (2.7) | 1 (2.4) | 1 (2.3) | 1 (3.6) | 0.741 |
|  | 1-6 months | 11 (9.8) | 4 (9.8) | 5 (11.6) | 2 (7.1) |  |
|  | 7-12 months | 16 (14.3) | 7 (17.1) | 8 (18.6) | 1 (3.6) |  |
|  | More than a year | 12 (10.7) | 5 (12.2) | 4 (9.3) | 3 (10.7) |  |
|  | Not able to define | 70 (62.5) | 24 (58.5) | 25 (58.1) | 21 (75.0) |  |
| **Currently used prophylactic medication: duration of use** | | | | | | |
| **Beta-blockers, N (%)**  Total 121/338 | Less than a month | 7 (5.8) | 2 (4.1) | 5 (10.2) | 0 (0.0) | 0.737 |
|  | 1-6 months | 22 (18.2) | 8 (16.3) | 8 (16.3) | 6 (26.1) |  |
|  | 7-12 months | 18 (14.9) | 6 (12.2) | 8 (16.3) | 4 (17.4) |  |
|  | More than a year | 19 (15.7) | 7 (14.3) | 8 (16.3) | 4 (17.4) |  |
|  | Not able to define | 55 (45.5) | 26 (53.1) | 20 (40.8) | 9 (39.1) |  |
| **Anti-epileptics, N (%)**  Total 97/338 | Less than a month | 3 (5.4) | 0 (0.0) | 2 (8.3) | 1 (6.7) | 0.687 |
|  | 1-6 months | 9 (16.1) | 1 (5.9) | 4 (16.7) | 4 (26.7) |  |
|  | 7-12 months | 13 (23.2) | 5 (29.4) | 5 (20.8) | 3 (20.0) |  |
|  | More than a year | 5 (8.9) | 2 (11.8) | 3 (12.5) | 0 (0.0) |  |
|  | Not able to define | 26 (46.4) | 9 (52.9) | 10 (41.7) | 7 (46.7) |  |
| **Anti-depressants, N (%)**  Total 56/338 | Less than a month | 8 (8.2) | 2 (6.9) | 5 (10.6) | 1 (4.8) | 0.155 |
|  | 1-6 months | 15 (15.5) | 5 (17.2) | 10 (21.3) | 0 (0.0) |  |
|  | 7-12 months | 16 (16.5) | 4 (13.8) | 6 (12.8) | 6 (28.6) |  |
|  | More than a year | 11 (11.3) | 6 (20.7) | 3 (6.4) | 2 (9.5) |  |
|  | Not able to define | 47 (48.5) | 12 (41.4) | 23 (48.9) | 12 (57.1) |  |
| **Onabotulinum toxin A,**  **N (%)**  Total 43/338 | Less than a month | 1 (2.3) | 0 (0.0) | 0 (0.0) | 1 (4.5) | 0.517 |
|  | 1-6 months | 18 (41.9) | 1 (25.0) | 9 (52.9) | 8 (36.4) |  |
|  | 7-12 months | 9 (20.9) | 0 (0.0) | 3 (17.6) | 6 (27.3) |  |
|  | More than a year | 5 (11.6) | 0 (0.0) | 2 (11.8) | 3 (13.6) |  |
|  | Not able to define | 10 (23.3) | 3 (75.0) | 3 (17.6) | 4 (18.2) |  |
| **Any other, N (%)**  Total 78/338 | Less than a month | 8 (10.3) | 3 (10.3) | 3 (9.4) | 2 (11.8) | 0.913 |
|  | 1-6 months | 11 (14.1) | 3 (10.3) | 7 (21.9) | 1 (5.9) |  |
|  | 7-12 months | 6 (7.7) | 2 (6.9) | 2 (6.2) | 2 (11.8) |  |
|  | More than a year | 12 (15.4) | 4 (13.8) | 5 (15.6) | 3 (17.6) |  |
|  | Not able to define | 41 (52.6) | 17 (58.6) | 15 (46.9) | 9 (52.9) |  |
| **Number of currently used medications** | |  | | | | |
| **Acute medications, N (%)** | 1 | 57 (18.0) | 28 (21.9) | 21 (16.5) | 8 (13.1) | 0.119 |
|  | 2 | 153 (48.4) | 68 (53.1) | 60 (47.2) | 25 (41.0) |  |
|  | 3 | 95 (30.1) | 29 (22.7) | 42 (33.1) | 24 (39.3) |  |
|  | 4 or more | 11 (3.5) | 3 (2.3) | 4 (3.1) | 4 (6.6) |  |
| **Prophylactic medications, N (%)** | 1 | 193 (68.9) | 82 (80.4) | 74 (63.8) | 37 (59.7) | **0.018** |
|  | 2 | 64 (22.9) | 15 (14.7) | 32 (27.6) | 17 (27.4) |  |
|  | 3 | 19 (6.8) | 5 (4.9) | 9 (7.8) | 5 (8.1) |  |
|  | 4 | 4 (1.4) | 0 (0.0) | 1 (0.9) | 3 (4.8) |  |
| **Currently used medication monotherapies and combinations** | | | | | | |
| **Acute medication, N (%)** | Non-opioid pain relievers & triptans | 106 (33.5) | 46 (35.9) | 47 (37.0) | 13 (21.3) | **0.014** |
|  | Non-opioid pain relievers, triptans & anti-emetics | 66 (20.9) | 24 (18.8) | 29 (22.8) | 13 (21.3) |  |
|  | Triptans only | 29 (9.2) | 10 (7.8) | 12 (9.4) | 7 (11.5) |  |
|  | Non-opioid pain relievers, triptans & other (not specified) | 20 (6.3) | 4 (3.1) | 10 (7.9) | 6 (9.8) |  |
|  | Non-opioid pain relievers only | 19 6.0) | 12 (9.4) | 7 (5.5) | 0 (0.0) |  |
|  | Non-opioid pain relievers & anti-emetics | 15 (4.7) | 6 (4.7) | 7 (5.5) | 2 (3.3) |  |
|  | Other combinations | 61 (19.3) | 26 (20.3) | 15 (11.8) | 20 (32.8) |  |
| **Prophylactic medications, N (%)** | Beta-blockers only | 66 (23.5) | 34 (33.0) | 25 (21.6) | 7 (11.3) | **0.002** |
|  | Other (not specified) | 55 (19.6) | 25 (24.3) | 19 (16.4) | 11 (17.7) |  |
|  | Anti-depressants only | 35 (12.5) | 16 (15.5) | 14 (12.1) | 5 (8.1) |  |
|  | Beta-blockers & anti-depressants | 24 (8.5) | 7 (6.8) | 11 (9.5) | 6 (9.7) |  |
|  | Anti-epileptics only | 23 (8.2) | 7 (6.8) | 11 (9.5) | 5 (8.1) |  |
|  | Onabotulinum toxin A only | 15 (5.3) | 1 (1.0) | 5 (4.3) | 9 (14.5) |  |
|  | Other combinations | 63 (22.4) | 13 (12.6) | 31 (26.7) | 19 (30.6) |  |
