# Supplementary material for: My Migraine Voice survey: disease impact on healthcare resource utilization, personal and working life in Finland
Source: J Headache Pain. 2020 Sep 29;21(1):118. doi: 10.1186/s10194-020-01185-4 (PMC7526198; doi:10.1186/s10194-020-01185-4)
Supplement: Supplementary file 5 — Additional file 5. Satisfaction and dissatisfaction in current acute and prophylactic medication and specific causes in these groups (prophylactic medication). [file 10194_2020_1185_MOESM5_ESM.docx]

**Additional file 5.** Satisfaction and dissatisfaction in current acute and prophylactic medication and specific causes in these groups (prophylactic medication).

|  | **Overall** | **4 ≤ MMD < 8** | **8 ≤ MMD < 15** | **MMD ≥ 15** | **p-value** |
| --- | --- | --- | --- | --- | --- |
| **Satisfaction in current medication** | | | | | |
| **Acute medication, N (%)** | | | | | |
| Fully satisfied | 37 (11.6) | 21 (16.4) | 10 (7.8) | 6 (9.7) | 0.477 |
| Somewhat satisfied | 182 (57.1) | 74 (57.8) | 74 (57.4) | 34 (54.8) |  |
| Neither satisfied nor dissatisfied | 42 (13.2) | 15 (11.7) | 18 (14.0) | 9 (14.5) |  |
| Somewhat dissatisfied | 49 (15.4) | 15 (11.7) | 22 (17.1) | 12 (19.4) |  |
| Fully dissatisfied | 9 (2.8) | 3 (2.3) | 5 (3.9) | 1 (1.6) |  |
| **Prophylactic medication, N (%)** | | | | | |
| Fully satisfied | 23 (8.2) | 17 (16.5) | 3 (2.6) | 3 (4.8) | **0.027** |
| Somewhat satisfied | 102 (36.3) | 36 (35.0) | 47 (40.5) | 19 (30.6) |  |
| Neither satisfied nor dissatisfied | 69 (24.6) | 22 (21.4) | 30 (25.9) | 17 (27.4) |  |
| Somewhat dissatisfied | 53 (18.9) | 19 (18.4) | 20 (17.2) | 14 (22.6) |  |
| Fully dissatisfied | 34 (12.1) | 9 (8.7) | 16 (13.8) | 9 (14.5) |  |
| **Reasons for satisfaction in current prophylactic medication** | | | | | |
| N (completely + somewhat satisfied) | 125 | 53 | 50 | 22 |  |
| Reduces number of migraine attacks | 75 (60.0) | 41 (77.4) | 21 (42.0) | 13 (59.1) | **0.001** |
| Easy to use | 67 (53.6) | 31 (58.5) | 28 (56.0) | 8 (36.4) | 0.197 |
| My quality of life has improved | 61 (48.8) | 26 (49.1) | 24 (48.0) | 11 (50.0) | 0.987 |
| Reduces my symptoms significantly | 56 (44.8) | 24 (45.3) | 22 (44.0) | 10 (45.5) | 0.989 |
| Reduces the intensity of my symptoms | 56 (44.8) | 17 (32.1) | 26 (52.0) | 13 (59.1) | **0.042** |
| Helps me control my migraine | 49 (39.2) | 19 (35.8) | 22 (44.0) | 8 (36.4) | 0.668 |
| Reduces my symptoms partially | 48 (38.4) | 13 (24.5) | 27 (54.0) | 8 (36.4) | **0.009** |
| Has few side-effects | 47 (37.6) | 18 (34.0) | 22 (44.0) | 7 (31.8) | 0.476 |
| Affordable | 41 (32.8) | 16 (30.2) | 18 (36.0) | 7 (31.8) | 0.816 |
| It helps me to deal with my migraine attacks | 30 (24.0) | 11 (20.8) | 14 (28.0) | 5 (22.7) | 0.729 |
| Mode of administration | 27 (21.6) | 9 (17.0) | 14 (28.0) | 4 (18.2) | 0.389 |
| Helps me get back to my daily activities | 24 (19.2) | 7 (13.2) | 11 (22.0) | 6 (27.3) | 0.301 |
| Has no side-effects | 19 (15.2) | 9 (17.0) | 6 (12.0) | 4 (18.2) | 0.718 |
| I am not afraid of the next attack | 18 (14.4) | 7 (13.2) | 7 (14.0) | 4 (18.2) | 0.890 |
| Other | 8 (6.4) | 2 (3.8) | 3 (6.0) | 3 (13.6) | 0.263 |
| Has desirable side-effects | 2 (1.6) | 0 (0.0) | 1 (2.0) | 1 (4.5) | 0.172 |
| **Reasons for dissatisfaction in current prophylactic medication** | | | | | |
| N (completely + somewhat dissatisfied) | 87 | 28 | 36 | 23 |  |
| It is not a cure | 51 (58.6) | 15 (53.6) | 25 (69.4) | 11 (47.8) | 0.208 |
| Too many side-effects | 39 (44.8) | 15 (53.6) | 16 (44.4) | 8 (34.8) | 0.405 |
| Does not relieve my symptoms at all | 31 (35.6) | 7 (25.0) | 15 (41.7) | 9 (39.1) | 0.354 |
| Relieves my symptoms partially | 23 (26.4) | 10 (35.7) | 6 (16.7) | 7 (30.4) | 0.202 |
| I do not want to take so many tablets/injections | 9 (10.3) | 5 (17.9) | 1 (2.8) | 3 (13.0) | 0.113 |
| Too expensive | 4 (4.6) | 0 (0.0) | 3 (8.3) | 1 (4.3) | 0.366 |
| Other | 4 (4.6) | 0 (0.0) | 2 (5.6) | 2 (8.7) | 0.292 |
| It is not reimbursed | 2 (2.3) | 0 (0.0) | 0 (0.0) | 2 (8.7) | 0.068 |
| I do not like the way in which I have to take my medication | 1 (1.1) | 0 (0.0) | 0 (0.0) | 1 (4.3) | 0.264 |
