# Supplementary material for: My Migraine Voice survey: disease impact on healthcare resource utilization, personal and working life in Finland
Source: J Headache Pain. 2020 Sep 29;21(1):118. doi: 10.1186/s10194-020-01185-4 (PMC7526198; doi:10.1186/s10194-020-01185-4)
Supplement: Supplementary file 6 — Additional file 6. Results from the negative binomial regression analysis. [file 10194_2020_1185_MOESM6_ESM.docx]

**Additional file 6.** Results from the negative binomial regression analysis.

| **Outcome** | **Variable** | **Level** | **Beta/Regression coefficient** | **exp(beta)** | **Lower 95% CI** | **Upper 95% CI** | **p-value** |
| --- | --- | --- | --- | --- | --- | --- | --- |
| HCP visits | Number of comorbidities | - | 0.08 | 1.08 | 0.02 | 0.14 | **0.009** |
|  | MMD | - | 0.04 | 1.04 | 0.01 | 0.07 | **0.001** |
|  | Gender (ref. Male) | Female | 0.08 | 1.08 | -0.46 | 0.57 | 0.757 |
|  | Age (years) | - | -0.01 | 0.99 | -0.02 | 0 | 0.193 |
| ER visits | Number of comorbidities | - | 0.07 | 1.08 | -0.01 | 0.16 | 0.110 |
|  | MMD | - | 0.07 | 1.07 | 0.03 | 0.11 | **<0.001** |
|  | Gender (ref. Male) | Female | 0.23 | 1.26 | -0.65 | 1.07 | 0.595 |
|  | Age (years) | - | -0.02 | 0.98 | -0.04 | -0.01 | **0.010** |
| Inpatient days | Number of comorbidities | - | 0.22 | 1.24 | 0.04 | 0.41 | **0.023** |
|  | MMD group | - | 0.09 | 1.1 | 0.01 | 0.18 | **0.020** |
|  | Gender (ref. Male) | Female | 0.41 | 1.5 | -1.6 | 2.11 | 0.671 |
|  | Age (years) | - | -0.06 | 0.94 | -0.11 | -0.02 | **0.002** |
